# Supplementary material for: An archaeomagnetic study of the Ishtar Gate, Babylon
Source: PLoS One. 2024 Jan 17;19(1):e0293014. doi: 10.1371/journal.pone.0293014 (PMC10793895; doi:10.1371/journal.pone.0293014)
Supplement: Attachment — Submitted filename: PLOS_Reply_27October.docx [file pone.0293014.s001.docx]

October 24^th^ 2023

Dear PLoS ONE editor,

We were happy to follow up on your and the reviewers’ comments and we hereby submit a revised manuscript. Below, please find our specific responses to the comments.

We hope the paper will be accepted for publication and thank you very much for your efforts in the publication process,

Lisa Tauxe, on behalf of all co-authors

**Journal Requirements:**

In your manuscript, please provide additional information regarding the specimens used in your study. Ensure that you have reported human remain specimen numbers and complete repository information, including museum name and geographic location.

*All relevant information is provided.*

If permits were required, please ensure that you have provided details for all permits that were obtained, including the full name of the issuing authority, and add the following statement:

'All necessary permits were obtained for the described study, which complied with all relevant regulations.'

If no permits were required, please include the following statement:

'No permits were required for the described study, which complied with all relevant regulations.'

*No permits were required; the relevant statement was added at the end of the manuscript.*

**Additional Editor Comments:**

This is an important contribution by the authors who have been involved in building the Levantine archaeomagnetic curve, which is becoming a necessary, precise tool for dating in the region. The current paper expands on previous work by the authors (leading scholars in the field of paleomagnetism) both geographically and methodologically in terms of the materials used. This will clearly be an important paper and worthy of publication in PlosOne, subsequent to minor revisions offered by 3 reviewers, as well as 2 additional comments I offer for the authors' consideration:

Lines 210-213: Although I agree with the caution on determining the construction of the gate as a result of the victorious siege over Jerusalem, this is almost mentioned in passing. I believe this discussion could be expanded, in considering the dating of the gate and in relation to other successful military accomplishments attributed to Nebuchadnezzar (the Battle of Carchemish, the conquest of Ashkelon and Ekron, the battle over Tyre).

*Thanks. This section has been revised and now includes more details (also see below).*

Lines 224-225: Significant advances have been made in the radiocarbon calibration, which when used together with micro-archaeological sampling and stratigraphy, allows for very narrow dating windows.

*Yet, we still have several periods of plateaus in the calibration curve – including during the 6^th^ c. BCE. We’ve added a note regarding this.*

**Reviewer #1**

The following notes pertain to the historical (Assyriological) aspects of the paper:

The mention of Nebuchadnezzar’s instructions to build the gate should be properly referenced. Footnote 28 directs the reader to Kaniuth 2018, but it lacks specific page references. Kaniuth (2018: 350) himself directs the reader to two (entire) books (Berger 1973 and Da Riva 2008), with no further specification (neither page nor text numbers are given). It would be more useful to refer the reader to Da Riva 2008: 118 § 2.1b, where the specific inscriptions are listed with references to Berger.

*We have now included a reference directly to the inscription. However, not to these two publications, since these are outdated in our opinion, but to the current online reference work.*

Regarding the statement: “it was even considered that the latest phase was constructed under Nebuchadnezzar II” (p. 10 (4/11), ll. 96–97); I suspect that what was meant is actually: "construction POST Nebuchadnezzar II," which would make more sense in this context; cf., l. 235, p. 14 (8/11), where the authors indeed use “post Nbk.”

*Thanks. The entire section has been revised.*

Concerning the three phases of the gate, since this paper is of interest to scholars from various backgrounds (the present reviewer is an Assyriologist, for example), it may be helpful to include a brief explanation of the nature of the three archaeological phases. It is important to explain to the reader the basis for distinguishing the different phases, such as different construction techniques, materials used, or the stylistic aspects of the glaze.

*We've added two more sentences and two more references so there's a little more context to the bricks now.*

Understandably, the identification of three phases raises questions about possible chronological gaps between them. It may also be worth mentioning, however, that (to the best of my recollection, without further investigation) no other king is known to have claimed responsibility for building the gate.

Regarding p. 13 (7/11), ll. 205–213: I do not have access to Fitzgerald/Knott 2019 (FN 43), so I could not follow the referenced claim (here too, parenthetically, no page numbers are given, just the book). Regardless, I am not familiar with the suggestion that the Ištar Gate was a “triumphal arch” erected to commemorate the 586 Jerusalem campaign. This notion appears peculiar and problematic (in its Babylonian context), seemingly resonating with Roman practices, Bibliocentrism, or both(?). Even if this argument is presented in/by Fitzgerald/Knott 2019, it would be advisable to provide further elaboration and, I would add, exercise caution in presenting such an assertion.

*We agree, and toned down the possible connection to the conquest of Jerusalem. We also changed the reference in regard to this issue.*

**Reviewer #2:**

This is important original pioneering research using the magnetic intensity for dating archaeological baked brick from the Ishtar Gate in ancient Babylon. The study is provisional with a lack of localized comparative results therefore instead relying essentially on the more established Levatine Archaeomagnetic Curve. As described in the paper, many baked bricks from the area have inscriptions allowing the historic date, and it should therefore be rather easy to continue with more analysis of bricks producing a local archaeomagnetic curve.

There are some historical and archaeological problem with the paper. The three phases of the gate discussed in the paper are the three types of wall decoration as presented in the museum and are not all the archaeologically attested levels of rebuilding the gate during the reign of Nebuchadnezzar II (605-562). The archaeological excavations as documented in R. Koldewey, 1918, Das Ischtar-Tor in Babylon, and summarized with updated material from cuneiform inscriptions by O. Pedersén in references [26] and [27] show a gate connected with the city wall rebuilt on much higher terrain several times during the 43-year reign of Nebuchadnezzar. He probably started the first version of the gate quite early in his reign, the first main rebuilding after raising the processional street through the gate several meters have a date 592 BC according to cuneiform texts. Later in his reign the street and gate were raised and rebuilt several times. The samples tested are probably all from rebuildings of the gate late in the reign of Nebuchadnezzar even if it is not said in the paper from where the used Phase I brick was taken.

*We have rewritten the corresponding passage and hope it is more understandable.*

The resultant date 569 BC is reasonable, but provisional until a local archaeomagnetic curve has been established. It disproves the suggestion in reference [28] that the latest phase was constructed after Nebuchadnezzar. Here the manuscript has a typing mistake: it says *under* and not the correct *after* Nebuchadnezzar.

*Fixed.*

The correct understanding of the subsequent rebuildings of the gate during the long reign of Nebuchadnezzar makes the discussions if the gate was built before or after the destruction of Jerusalem irrelevant as both are true. It would be better to take this a bit speculative part away before publication.

*We have toned down this possible connection.*

Would it be possible to name the lab where the test have been made?

*Added.*

**Reviewer #3:**

This article meets the quality standards for PLOS ONE. It addresses an interesting issue as the investigation of intensity changes for a region relatively unexplored so far, such as Southern Mesopotamia. Furthermore, the paper explores the chronological and historical implications of the results obtained. The new archaeomagnetic data have been obtained from very well-dated bricks following standard procedures and are of good quality. These new data provide unique information about past values in geomagnetic field intensity for this region and time interval. The subsequent analysis and conclusions drawn from the data are consistent and well-presented. The paper is well-written, well-organized and the quality of the figures is high. I therefore recommend publication after minor changes. I have, however, some concerns that should be addressed before the final publication:

• Lines 47-57. I would like to read a more detailed description of the quality of the data available for the region.

*We added a sentence about this citing the Shaar and Gallet papers regarding methods.*

• Line 56: This statement is not accurate. It is true that in western Europe, this peak does not appear to be present but a recent study by Rivero-Montero et al. (2021) demonstrated a high-intensity peak is Greece between 1070 and 1040 BCE, suggesting its association to the LIAA. Please, include this information in the new version.

*We added this now. thanks to the reviewer for alerting us to this publication.*

• Figure 2: the explanation of Figure 2 is missing (lines 105-106)

*The caption is in the manuscript. We are not sure what is the source of the confusion.*

• Lines 106-119. What about TRM anisotropy and cooling rate corrections? Have these effects been measured? Nothing is said about these important issues. I know that the TRM anisotropy´s effect on bricks is generally low, but some details about this should be included. On the other hand, it is not explained if the cooling rate has been measured or considered. If this was not the case, do you consider that your mean intensity should be affected? How would your results and derived dating be modified by, for example, a mean value of 5% for the cooling rate effect upon paleointensity estimates?

*We added an explanation about this - that ATRM was done (and it was trivial) and that cooling rate was not done as the companion paper on a larger set of bricks by Howland et al. (in press) shows that was also negligible in all of those Mesopotamian bricks.*

• Do you have an explanation for the higher dispersion observed in Figure 4 results? It seems that some of the results might be affected by some effects such as MD behaviour since the trend seems not to be linear. It would be nice to show additional NRM-TRM plots.

*As all data are in the MaGIC Database, these (and other) plots are readily available.*

• Lines 194-213: How reliable is the proposed date? If we take into account both the errors in the PSV reference curve used and the mean intensity of Ishtar Gate, an age interval is obtained instead of an exact date. An accurate archaeomagnetic dating anañysis should be included here, considering the uncertainties of both the curve and the data. Please, improve this section.

*The date and its errors were provided.*

• In general, the quality of the figures in the pdf file is not high, but I assume that they should be ok when submitted as individual files.

*We’ve submitted high resolution files, thanks.*

• I also recommend including references from other international groups devoted to archaeomagnetic research to enrich the context. While the provided references are relevant, incorporating these citations would strength the paper´s global perspective.

*We have in the manuscript basic references that include all the other relevant references for the archaeomag of this region.*

I hope it helps.

*Thank you*
